# The stress of nursing: exploring communicatively restricted organizational stress (CROS), effort-reward imbalance, and organizational support among a sample of U.S. working nurses

**DOI:** 10.1186/s12995-023-00390-6

**Published:** 2023-10-02

**Authors:** Justin P. Boren, Alice E. Veksler

**Affiliations:** 1 Department of Communication, Santa Clara University, 500 El Camino Real, Santa Clara, CA 95053-0277 USA; 2 Department of Communication, Christopher Newport University, Newport News, VA USA

**Keywords:** Nursing, Stress, Co-worker relationships, Healthcare settings, Health communication, Nurse relationships

## Abstract

**Background:**

Nurses experience a constellation of negative outcomes such as lost productivity, based on their high levels of organizational stress. Following recommendations for best practices in health communication can dramatically improve the organizational climate for nurses and can have a significant effect on patient outcomes. In this study, we evaluate the impact of Communicative Restricted Organizational Stress (CROS) and effort-reward imbalance (ERI).

**Methods:**

A mixed-methods approach was employed. A professional survey research vendor was contracted to obtain an appropriate national sample (*N* *=* 299) of working nurses in the United States of America. Participants completed an online closed-ended questionnaire for the quantitative portion of the study. Qualitative data were gathered from member-checking follow-up interviews.

**Results:**

Results of the quantitative analysis indicated that nurses experience CROS, that these experiences are distressing, that CROS functions as an effort in the effort-reward-imbalance model, and that CROS and ERI contribute to negative outcomes such as insomnia, productivity lost, and poor general health. Specifically, a moderated moderation model accounted for 53% of the variance [*F* (7,290) = 47.363, *p* < .001] indicating that nurses with high levels of CROS distress and low levels of organizational support experienced the highest level of ERI in the presence of high nursing stress, *t* (296) *=* 3.05, *p* = .03, 95% CI [0.0038, 0.0178]. These findings were validated through member-checking qualitative interviews and specific overarching themes were explicated.

**Conclusions:**

CROS is an important variable in understanding the experience of nursing stress. Furthermore, CROS serves as an effort in the ERI Model and serves to exacerbate nursing stress. We recommend practical implications for the improvement of psychosocial stress in an occupational environment for nurses.

American workers have been reporting increasing amounts of stress even before the COVID-19 pandemic changed the landscape of work in America. In fact, based on a 2021 survey of 1500 workers, three in five workers reported that work-related stress impacts their lives in a negative way [1]. Among the occupations consistently ranked highest in reported stress were those in a healthcare setting [2]. Organizational membership for healthcare workers has long been associated with a wide variety of psychological stressors which can have a significant negative effect on their health and well-being [3, 4]. This in turn, can affect patient health and safety [2] and as such, is a major concern for both healthcare researchers and practitioners. Within the healthcare profession, nurse stress is strongly associated with issues of retention and turnover [2], which given the ongoing nursing shortage [5] makes this an issue of particular importance.

Stress is a complex phenomenon comprised of an individual’s psychological, physiological, and behavioral responses to real or perceived sources of strain [6, 7]. Chronic workplace stress places a strain on all aspects of health [6]. Workers under chronic stress risk burnout, report high amounts of depression and anxiety, and are under increased risk for post-traumatic stress disorder [4, 8]. Chronic stress leads to downregulation of the immune system, [9] metabolic syndrome, cardiovascular disease, [10] and worker mortality [11]. Given the specific demands of their jobs, healthcare workers (and nurses in particular) are especially vulnerable to stress and stress-related disorders [12–14].

Nurses comprise the largest number of all occupations in the health professions [5]. In fact, there is an estimated 3.9 million nurses and midwives in the US and nursing is expected to be the fastest growing field of employment in the US [5]. However, nursing turnover rates are estimated to be as high as 37% for certain specialties [5] and this is a major concern for the profession [2, 5, 15]. Specifically, nurses have been found to experience some of the most stressful working conditions as compared to other healthcare occupations leading to the largest amount of burnout [16]. Compassion fatigue, general stress, as well as concerns about safety and wellness, are just a few of the issues nurses must deal within their work environments [2]. Although much work has been done to examine the various sources of stress for nurses, both before and during the pandemic [2, 17–19], stress-mitigating and stress-exacerbating features of the workplace for nurses require additional explication. For instance, current models such as effort-reward imbalance, have been used to start to demonstrate how areas of reward can function to offset the stressors nurses experience [20].

The effort-reward imbalance (ERI) model is an explanatory stress-focused model of organizational environments [21]. The model describes the relationship between the stressful elements of one’s work compared to the perceived rewards received from that work. ERI “asserts that the recurrent experience of failed reciprocity between high cost spent at work and low gain received in turn, activates sustained negative emotions of reward frustration and associated circuits of the brain reward system” [21]. ERI is firmly rooted in a social-exchange perspective, whereby excess rewards can compensate for greater efforts, but increasing efforts tend to weaken the perception of the relationship. In the case of ERI, the relationship is between the worker and their perception of the workplace environment. According to the model, efforts include all demands and obligations (physical, psychological, psychosocial, etc.) and rewards include adequate remuneration, esteem, job security, and social relationships, among others. When the perceived efforts of the job outweigh the rewards, the worker perceives an imbalance, which is inherently stressful [22, 23]. However, we contend that to date, such ERI examinations fail to adequately explain how the experience of stress relates to relevant outcomes. Specifically, for nurses, what happens when they experience a meta-stressor that compounds their perceived “efforts?”

One such meta-stressor is Communicatively Restricted Organizational Stress (CROS) which plays an important role in the way that stress is experienced and exerts profound negative effects on the stressed individual’s lived outcomes [24]. When a person experiences CROS, they feel limited or frustrated in their ability to discuss their stressors with others, acting as a meta-stressor for that individual (i.e., stress relating to the stress of not being able to communicate). As a result, the experience of the stressor becomes amplified [24, 25]. CROS functions in multiple ways:

First, the experience of CROS decreases an individual’s ability to directly address and/or resolve the stressor. Second, CROS can be experienced as a lack of social support if an individual feels that he or she has few (if any) people to turn to for help. Next, CROS can be experienced as a decrease in coping ability if one’s perception of available support is reduced. Finally, CROS can frustrate one’s ability to convert perceived support into received support [24].

We believe that CROS is especially prevalent among nurses given the specialized nature of their work and the institutional and legal barriers such as HIPAA that prevent open dialogue about their work experiences. Further, we propose that the presence of CROS is an effort in the workplace for nurses. Nurses would need to expend more energy to locate support networks, endeavor to communicate about their support needs to others, and navigate the complexities of attempting to communicate with others. When nurses are distressed by their restricted ability to communicate about stressors, this distress then becomes an added effort at work. Therefore, our belief is that CROS can be a potential effort in an organizational context. We believe this to be especially true, given that available social support is viewed as a reward in ERI [21].

Therefore, the purpose of this paper is to examine the effects of CROS as it interacts with organizational stress among working nurses. Data were collected prior to the start of the COVID-19 public health emergency in the United States of America, and as such, our findings reflect the impact of long-standing systemic issues associated with working in nursing, independently of newly introduced COVID-19 related stressors. To collect our data, we conducted a mixed-methods approach, which included a quantitative closed-ended survey collected through a nationally representative survey of working nurses across areas of specialties and follow-up qualitative interviews. First, we present a mediated moderation model illustrating how CROS is associated with the ERI model of workplace stress. Next, we present findings from a member-checking interview procedure, which allows us to contextualize the results. As such, this project presents a novel approach to understanding how and why stress can be so pervasive among nurses and we present some implications and suggestions for improving work conditions for this population.

## Research questions and hypotheses

Given the research on CROS, evidence suggests that many (although not all) members of organizations experience some amount of CROS [24]. However, no research has focused specifically on nurses; therefore, we aim to identify the extent to which CROS exists for this specific population. We acknowledge however, that not all nursing specialties function the same way, and therefore we cannot assume nurses are homogenous. We endeavor, then, to delineate how different nurses experience stress. With those goals in mind the following research questions are proposed:

- RQ_1_: What is the prevalence of CROS among nurses?
- RQ_2_: How does CROS distress differ by nursing type?
- RQ_3_: How does nursing stress differ by nursing type?

Past research on CROS allows us to make some predictions about how we expect CROS to function. Specifically, given that we conceptualize CROS as an effort, we would expect that CROS should correlate positively with ERI Imbalance. Furthermore, given that CROS and ERI imbalance in other contexts have been associated with negative individual level outcomes [20, 21, 24, 26], we would expect the same to be true for nurses. Therefore, we propose the following principal hypotheses:

- H_1_: Among nurses, ERI is positively correlated with perceived productivity lost and insomnia and negatively correlated with general health.
- H_2_: Among nurses, CROS distress moderates the relationship between nursing stress and ERI which is moderated by perceived organizational support, such that those with high levels of CROS distress and high levels of nursing stress with the lowest level of organizational support will have the highest reported amount of ERI.

By demonstrating that CROS contributes to the experience of nurse stress, simple interventions can be designed to alleviate this unique exacerbator of the stress experience. As a result, it may be possible to alleviate some of the pressures on nurses, decrease their burnout, reduce turnover, and improve health and well-being for both nurses and their patients. Given the importance of these goals, our aim is to empirically identify the presence of CROS among nurses and evaluate the extent to which it affects overall health and other related outcomes. Furthermore, we seek to demonstrate how CROS fits within an ERI framework to help explain nurses’ experiences of workplace stress.

## Methodology

Our primary methodology was to collect data from working nurses with a nationally representative survey technique using a closed-ended questionnaire. We then conducted follow-up member-checking interviews with some of the participants who completed the questionnaire. The entire study protocol was reviewed and approved by both authors’ institutional review boards.

### Recruitment strategy

We contracted with Qualtrics, Inc. (a professional survey company located in Seattle, WA), to recruit a panel of nursing professionals working in the USA, who were compensated by Qualtrics for their participation. Data were collected prior to the declaration of the COVID-19 emergency. Qualtrics utilized multiple data-sources to solicit nurses for recruitment. After consenting and verifying age (over 18) and employment in a nursing field, participants were able to proceed with the questionnaire. In consultation with Qualtrics, we employed data quality tests (screeners, attention filters, completion time analysis, and straight-lining analysis) and ended up excluding 13 people. Our final dataset included 299 individuals working in a nursing-related field distributed among 45 out of the 50 U.S. states. At the conclusion of the survey, respondents were given the option to volunteer to participate in the follow-up interview protocol with an added incentive of a $50 gift card.

### Measures

Descriptive statistics, reliability assessment, and zero-order correlations for all measures are reported in Table 1. All measures reported here had good internal consistency.

**Table 1:** Pearson’s Product Moment Correlation Coefficient Matrix for DVs (*N* = 299)

Variable	*M*	SD	1	2	3	4	5	6
1. CROS Distress	4.42	1.40	--					
2. ERI Ratio	1.25	0.55	0.30**	--				
3. Organizational Support	3.14	0.87	− 0.196**	− 0.65**	--			
4. Productivity Lost	19.67	21.35	0.02	0.195**	− 0.21**	--		
5. Insomnia	11.97	6.47	0.22**	0.33**	− 0.32**	0.27**	--	
6. Perceived General Health	3.26	0.96	− 0.17**	0.13*	0.18**	− 0.10*	− 0.397**	--

* *p* < .05. ** *p* < .01. Significance reported at the one-tailed level

#### Communicatively restricted organizational stress (CROS)

To evaluate CROS, we utilized Veksler and Boren’s [25] two-dimensional measure of CROS prevalence and distress (CROS-14). The CROS prevalence dimension contains six items and the distress dimension includes eight items, each on a Likert-type scale from 1 to 7 (strongly disagree to strongly agree) with higher amounts indicating more reported prevalence or distress of CROS, respectively. Participant scores are reported as means, making the range for each dimension of CROS, 1–7. Consistent with the measure’s instructions, we computed a mean score (after first recoding the reversed items) for both dimensions separately. The measure was deemed to be valid in the prior validation study and our reliability coefficients were acceptable, given the research context.

#### Effort-reward imbalance

Effort-Reward Imbalance [22, 27] is a stress-related model of organizational work which posits that chronic stress at work is due to high efforts spent with little reward received. We utilized the ERI Questionnaire Short Form [28], which probes this model through a 10-item questionnaire with four-point Likert-type statements (Strongly Disagree to Strongly Agree). There are three effort questions (e.g., “I have constant time pressure due to a heavy workload”) and seven reward questions (e.g., “I receive the respect I deserve from my superior or a respective relevant person”). The measure has been used extensively and has high validity, given its use in a variety of contexts. The measure has high discriminant and criterion validity and had similar statistical properties to the long-form version of the measure [28, 29].

A participant’s ERI was computed using a ratio formula, provided by the author of the measure (see [29]). The total score for a person’s effort is divided by their score for reward. That integer is then multiplied by a correction factor of 7/3 (to account for the seven reward items and three effort items). This yields a ratio, whereby a score of one represents “the person reports one effort for one reward” [29] a score of greater than 1 means that “the person reports more efforts for each reward” (p. 3), and less than one means that “there are less efforts for each reward” (p. 3). In either the higher- or lower-than-one condition, imbalance occurs. For our sample, ERI scores ranged from 0.28 to 4.00 (*M* = 1.24, *SD* = 0.55).

#### Perceived organizational support

Perceived Organizational Support (POS: [30]) was measured using an eight-item, unidimensional, Likert-style questionnaire (strongly disagree to strongly agree). The items tap into a participant’s belief that their organization supports them. The measure has been used in a variety of research studies and is both well-respected and parsimonious. The original validation study demonstrated good internal consistency, high factor validity, and high construct validity. For our sample, we computed an average score among the items for each participant and used that score in all subsequent analyses (*M* = 3.14, *SD* = 0.87). Higher numbers indicated more perceived support.

#### Nursing stress

To evaluate the stressors that nurses face, we used the Nursing Stress Scale (NSS: [31]), a widely used assessment tool for evaluating specific nursing stress, which presents 34 potentially stressful nursing situations. Participants indicated how often they found each situation stressful on a four-point scale from never to very frequently. The measure includes seven factors: Death and dying, conflict with physicians, inadequate preparation, lack of support, conflict with other nurses, workload, and uncertainty concerning treatment.

Based on the validation studies [31], the NSS has high test-retest reliability and had construct and criterion-linked validity. The NSS also had high predictive validity (insofar as predicting turnover rates among nurses). We believe that the NSS is the most appropriate measure, given these reasons. Following the authors’ recommendations, we utilized a composite sum score to calculate a nurse’s total stress with the “never” point in the measure being coded with a zero and the “very frequently” anchor being coded with a three. This yielded a potential NSS score of between 0 and 102 (for our study, *M* = 35.42, *SD* = 16.76, Range = 0–91).

#### Insomnia

To evaluate insomnia, we used the Insomnia Severity Index (ISI: [32]), a tested and validated measure of a person’s perception of how bad their insomnia is. The ISI has been used in both clinical and research applications and has high internal consistency and concurrent validity. The measure probes severity of insomnia problems over the past two weeks and asks respondents to rank their severity on seven items from mild (0) to very severe (4). The measure rates satisfaction with current sleep patterns (from very satisfied to very dissatisfied), how much sleep patterns interfere with their daily functioning, and how noticeable to others the problem is (not at all to very much). Finally, respondents indicate how worried or distressed their sleep patterns are to them (not at all to very much). Scoring is additive with clinical indicators of 0–7 (no clinical insomnia), 8–14 (subthreshold insomnia), 15–21 (clinical insomnia), and 22–28 (severe clinical insomnia). For our sample, scores ranged from 0 to 28 (*M* = 11.96, *SD* = 6.47).

#### Perceived diminished productivity and general health

To evaluate a nurse’s perception of their diminished productivity we followed the procedure in Daley et al. [33]. Participants were asked to indicate “over the past three months, by what proportion (if any) do you believe that your productivity at work has been diminished” on a scale of 0% (no loss) to 100% (total loss). That score was then used in subsequent analyses (*M* = 19.67, *SD* = 21.35). For general health, we used the single item “F1” perception question from the Health Information National Trends Survey (HINTS: [34]), which states “In general, would you say your health is…” and gives the options “Excellent,” “Very Good,” “Good,” “Fair,” or “Poor.” (p. 8). We recoded these items so that poor was scored with a one and excellent was scored with a five. Based on that, our sample had an average score of 3.26 (*SD* = 0.96), which is slightly higher than “Good.”

### Quantitative analysis

The research questions and hypothesis one were evaluated using descriptive analysis and univariate statistical techniques, including ANOVA and Pearson’s Product Moment Correlations. To test hypothesis 2, we evaluated the model holistically as a conditional process effect utilizing the PROCESS Macro in SPSS by conducting a moderated moderation analysis [35]. Each variable was mean-centered prior to analysis to guard against multicollinearity. A contrast analysis was conducted to illustrate the varying levels of any detected moderation [35] with a low level of organizational support and a high level of CROS (as defined as -1SD and + 1SD, respectively) versus the high level of organizational support and low level of CROS distress. For all analyses, any missing data were treated with pairwise deletion.

### Interview protocol

For additional context and to clarify some of the findings, we reached out to our participants for a 30-minute-long recorded interview at the conclusion of the survey. From the self-identified list of interested participants, we selected 10 nurses at random via email and set up a time to conduct the interviews. Semi-structured interviews were conducted via Zoom and recorded for audio with both investigators being present for each interview. Each participant was provided a separate consent and provided a pseudonym prior to recording and was offered a $50 gift card.

Utilizing our active notes during the meeting and a review of the Zoom generated transcript, both authors engaged in a thematic analysis process [36, 37] to allow latent themes to emerge through our reading of the member-checking interviews. We utilized the six-step process outlined in past literature which involved becoming comfortable with the whole corpus of data, generating initial codes, identifying themes, reviewing themes, defining the themes, and completing the final analysis. We generated codes inductively, allowing them to emerge from the data. Both authors then met to discuss their independent analyses and construct a codebook. Discrepancies between the two authors’ interpretations of the data were minimal and resolved through consultation. In the second phase of coding, the second author utilized the codebook to review the primary data to identify patterns and to begin the thematic identification process. Both authors then met to fully engage with the coding to identify the relationships between the themes and to draw conclusions from the data [36, 38, 39]. The flexibility of thematic analysis as a method allowed us to use this technique in the context of the post-positivist approach to this project [39].

## Results

### Participants

Survey participants included 278 female-identified (93%) and 16 male-identified (5.4%) people. Participant ages ranged from 20 to 80 years old (*M* = 41.56 years, *Mdn =* 39, *SD* = 11.95). These respondents had been employed at their current jobs for an average of 8.66 years (*Mdn* = 6, *SD* = 8.29) and worked an average of 37.35 h per week (*Mdn =* 40, *SD* = 12.37). Our sample was predominately “White, not Hispanic” (*n* = 245) with other ethnicities reported as being “Black or African American” (*n* = 36), “Hispanic or Latino/a” (*n* = 13), and “Asian/Filipino/Asian Indian” (*n* = 5). Highest education obtained included 98 (32.8%) with baccalaureate degrees, 96 (32.1%) with associate degrees, 58 (19.4%) with high school diplomas, 35 (11.7%) with master’s degrees, three with doctorates, and two with less than a high school degree. Most of the sample (*n* = 264) were regular employees (with only a few being employed through a travel nurse agency [*n* = 9], self-employed [*n* = 8], or employed through a temporary agency [*n* = 6]). Nursing Positions and Clinical Areas are reported in Table 2.

**Table 2:** Demographic Information for Sample of Nurses (*N* = 299)

Nursing Credential or Licensure	*n*	%
Registered Nurse	148	49.5
Certified Nursing Assistant	89	29.8
Vocational Nursing License (LPN/LVN)	48	16.1
Nurse Practitioner	13	4.3
Advanced Practice Registered Nurse	9	3.0
Settings for Principal Nursing Position	*n*	%
Hospital, inpatient care or emergency department	88	29.4
Home health agency/ home health service	45	15.1
Nursing home, extended care, or skilled nursing facility	39	13.0
Medical practice, clinic, physician office, surgery center	26	8.7
Hospital, ambulatory care department (surgical, clinical)	18	6.0
Hospital, nursing home unit	17	5.7
Inpatient mental health / substance abuse	10	3.3
Other type of position not listed	10	3.3
Hospital, ancillary unit	9	3.0
Clinical Areas for Principal Nursing Position	*n*	%
Geriatrics	43	14.4
Home Health Care	37	12.4
Emergency / Trauma	31	10.4
Other area not listed	22	7.4
Work in multiple areas and/or do not specialize	19	6.4
Not involved in direct patient care	17	5.7
Medical-Surgical	16	5.4
Ambulatory / Outpatient	13	4.3
Oncology	11	3.7
Pediatrics	11	3.7
Psychiatry / Mental Health	11	3.7
Rehabilitation	10	3.3

For space purposes, we have reported only the settings and clinical areas with a frequency greater than 3% in the sample. A full list is available from the authors

### CROS prevalence among nurses (RQ1)

The first research question asked about the prevalence of CROS among nurses. Exploring the descriptive statistics of CROS Prevalence in this sample revealed an average prevalence slightly higher than the hypothetical midpoint of the scale (a 1–7 scale with a 3.5 hypothetical midpoint), *M* = 3.60, *Mdn* = 3.67, Mode = 4, *SD* = 1.36, Range = 6. The scores represented a relatively normal distribution with unremarkable skewness and kurtosis.

### CROS distress and nursing stress by nursing type (RQs 2 & 3)

Table 3 reports the means and standard deviations for both RQ 2 and 3. For both research questions, we simplified the analysis by collapsing nursing type into seven distinct categories. Research question 2 asked about how CROS distress differs by nursing type. ANOVA results indicated a significant overall effect, *F* (6, 287) = 2.64, *p* = .017, η^2^ *=* 0.052 [0.002, 0.091]. Least Squared Difference post-hoc testing indicated that mental health nurses report the highest amounts of CROS distress, which significantly differed from all categories except medical practice nurses. Home health care nurses reported the lowest amount of CROS distress.

**Table 3:** Means (SD) of CROS Distress and Nursing Stress by Nursing Type for RQS 2 & 3

	Hospital (*n* = 115)	Nursing Home, Rehab., Hospice (*n* = 64)	Home Health Care (*n* = 45)	Medical Practice (*n* = 28)	Mental Health (*n* = 11)	Occupational & School Health (*n* = 11)	Other (*n* = 20)
CROS Distress	4.35 (1.41)	4.21 (1.37)	4.14 (1.33)	5.01 (1.34)	5.43 (1.43)	4.17 (1.48)	4.71 (1.37)
Nursing Stress	36.68 (15.74)	39.20 (16.48)	30.47 (17.60)	28.61 (13.54)	48.18 (22.09)	31.09 (23.43)	33.15 (12.61)

Research question 3 asked about how nursing stress differs by nursing type. ANOVA results indicated a significant overall effect, *F* (6, 287) = 3.47, *p* = .003, η^2^ *=* 0.068 [0.010, 0.112]. Least Squared Difference tests indicated that mental health nurses reported the highest amount of nursing stress and that difference was significant across all categories except for nurses working in nursing homes with medical practice nurses reporting the lowest levels of nursing stress.

### Hypotheses

We report all variable correlation coefficients in Table 1. The first hypothesis posited that ERI (*M* = 1.25, *SD* = 0.55) would be positively associated with productivity lost (*M* = 19.67, *SD* = 21.35) and insomnia (*M* = 11.97, *SD* = 6.47) and negatively correlated with general health (*M* = 3.26, *SD* = 0.96). The bivariate correlation analysis indicated that each of these variables were correlated in the predicted manner (*p* < .05). Therefore, hypothesis one is supported.

**Fig. 1:**
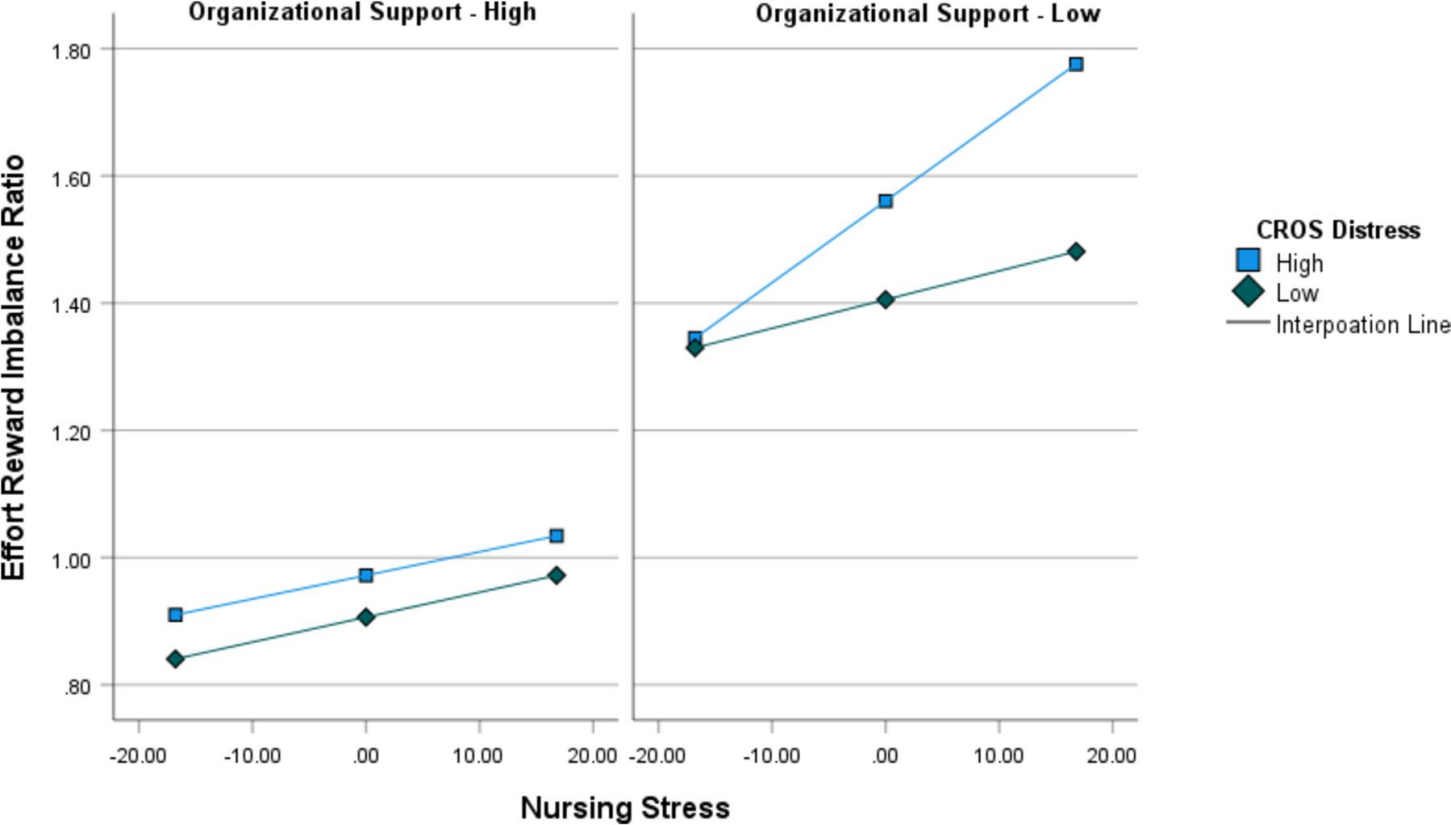
Visualization of Conditional Moderation Effect for Hypothesis 2. Note. This figure represents the conditional moderation effect for hypothesis 2 by plotting the +1SD (high) and -1SD (low) results for the moderators Perceived Organizational Support and CROS Distress of the predictor (Nursing Stress) on the criterion (Effort Reward Imbalance Ratio)

For hypothesis two, we hypothesized that the association between nursing stress and effort-reward imbalance would be moderated by perceived organizational support. Further, perceived organizational support would be moderated by CROS Distress. The overall model was significant, *F* (7,290) = 47.363, *p* < .001, *R* ^2^ = 0.53. Each of the variables in the model produced significant coefficients, although cross-product terms were not significant (indicating no primary interaction effects). The hypothesis did propose a conditional moderation effect such that those with high levels of CROS distress and low levels of organizational support will experience the highest level of ERI in the presence of high nursing stress. The result indicated that the conditional effects of X on Y were as predicted and significant, *t* (296) *=* 3.05, *p* = .03, 95% CI [0.0038, 0.0178]. In fact, those reporting low levels of organizational support with high levels of CROS had the highest reported level of Effort Reward Imbalance when nursing stress was high (see Fig. 1 for an illustration). Therefore, hypothesis 2 was supported.

### Qualitative results

The interview participants (*n* = 10) aged in range from 28 to 60 (*M* = 47.4, *SD* = 21.14) years and worked for their current employer for an average of 12.85 years (*SD* = 10.79). Five worked in a hospital or inpatient facility, three worked in a nursing home or skilled nursing facility, one was a home health nurse, and one worked for a state-run facility. All had direct patient contact. Clinical specialties included Emergency/Trauma (*n* = 2), Geriatrics (*n =* 2), surgery (*n* = 2), home health care, rehabilitation, medical-surgical, and ambulatory/outpatient.

A few themes emerged from our analysis of these interviews. First, the experience of stress for working nurses is universal and significant, and second, many of the nurses we spoke to felt restricted in their ability to discuss their stress (CROS). A third theme emerged, which was related to these nurses’ subjective experience of ERI. Among these 10 nurses, we noticed a pattern wherein the nurses who experienced an imbalance in their experience of stress vs. reward resulting from their work, also talked about significant CROS. Below, we articulate these themes and the codes nested within them.

#### Experience of stress as universal

##### Workload

Nearly every nurse we spoke with described how workload becomes a major stressor. They all described unreasonable amounts of work, little direct contact with patients due to that workload, and high turnover rate were primary stressors for their work. They also described issues relating to not getting adequate breaks, difficulty with work/life balance, and little agency to change or improve on any of these issues. For instance, Lindsey who works as a floor/charge nurse supervising the nurses and CNAs at a skilled nursing facility states:

*The facility I’m currently at, the ratio of nurse to patient is one to 18 or 19. Which is pretty high to begin with. That’s a lot of people to manage through the day and then call offs are frequent, and turnover is so frequent. And CNAs just get overwhelmed and then it makes everyone else overwhelmed and it’s just hard. I think that’s the most difficult part of it for me.*

##### Communication

The nurses in our sample also reported poor communication or lack of coordination with co-workers, lack of respect from physicians and managers, lack of communicated support from management, and prejudice or bullying from co-workers as significant stressors. For instance KD, an operating room nurse described their relationship with management about a particular issue:

*I did go to administration…administration did not do anything and the director of nursing just hammered me some more. She harassed me in front of my co-workers and she made my life a living hell really in the work environment, getting me to quit.*

Willow, a long-term care nurse, described this tension with her supervisors in this way:

*We feel that we don’t have respect, and that’s a big problem in nursing… It comes down from the director of the facility all the way down to the supervisors.*

Zelda who works in peri-anesthesia at a teaching hospital points out that communication between physicians and residents can be disconfirming, and lead to stress:

*And then you know, we can run into the doctors who don’t, um, see us as anything more than pill pushers or, you know, as an integral part of the healthcare team. We spend the time. We spend the bulk of the time with the patients. They don’t.*

In some cases, communication issues were more systemic insofar as nurses did not have efficient protocols for communicating information to supervisors or subordinates or from one shift to another. Nearly every nurse we spoke with described communication issues as stressful, especially when it meant that they were prevented from effecting institutional changes.

##### Bureaucracy

Nearly universal among our nurses, administrative bureaucracy was a noted stressor. Namely, participants reported a lack of support, unreasonable amounts of paperwork, unclear or burdensome policies, lack of resources, and an onerous focus on arbitrary performance metrics. For instance, Orchid, who works as a home health nurse and has worked as a floor nurse in a skilled nursing acute care facility says:

*Some of the stress comes from not having what you need to complete your job. And that could be anything from supplies to knowledge. Because for instance, one of my co-workers… she’s a new nurse and they had her doing something she hadn’t been trained for in. And I’m like, when I want back to the facility because I was working in a place for the intellectually disabled. So we didn’t have to do a lot of stuff. Like I didn’t know how to make some of the IV medications. I had to YouTube. And to me, this should be yeah… and this nurse had the same thing, she didn’t know how to do lymphedema labs so she had to YouTube… The biggest thing is we need the stuff to do our jobs. And I guess the supplies… I was at a facility once where we ran out of alcohol swabs and someone had to run to the CVS and buy a bunch of alcohol swabs. Yeah, and I think, like that shouldn’t happen and adds to the stress.*

##### Patients and families

Finally, we heard from nurses that patients themselves and their family members become a universal source of stress. Nurses reported that it was stressful to not be able to follow up with patients to know their outcomes, that it was hard to deal with “frequent flier” patients or medical misuse (e.g., non-emergency patients coming to the ER regularly), harassment or violence, and unrealistic expectations from patients and/or their families. For example, Lois talked about significant issues with patients:

*We have people that are assaulted. Like the other day at work. I was called a “See you next Tuesday.” The guy was completely…I mean, he was hammered. But, I mean, there was no reason for it. We’ve had people that have been assaulted.*

Similarly, other participants noted that family expectations can be unreasonable and when they are stressed and worried about their loved ones, they can be on edge and interactions with them can lead to added stress for the nurses. As expected, death of patients was also noted as a stressor although much less frequently than many of the other issues discussed above.

#### Experience of CROS

In an effort to try and make better sense of our quantitative data, we asked these nurses about their direct experience with CROS. These nurses had varying experiences with the extent to which they felt restricted in their ability to discuss their work stress. Some said that they had no difficulties speaking about their work stress. For instance, Maddy, an ER nurse who is also married to a nurse told us she can speak to her husband and did not feel a lot of CROS. A more common response from these nurses was that they only one single support outlet (e.g., co-workers or family) because the others were off-limits. Some nurses said that they were only comfortable speaking with peers whereas others felt speaking to peers was not appropriate and they would only speak to family or to supervisors.

Although some were comfortable speaking with supervisors or HR, others told us that they would be unlikely to do so. Nurses worried about futility of discussion, dismissiveness, and/or retribution when it came to speaking with supervisors or others above them. For example, Orchid stated:

But yeah I do talk it over because you always want to second guess yourself if something doesn’t go exactly as planned. And if it feels good to get, you know, to hear somebody say: no you know, you did the same thing I would have done, you did fine. So yeah I do talk it over with coworkers. I don’t necessarily talk it over with management, but I will talk it over with coworkers…. I guess because as much as you want to know if you did something wrong… you don’t really want to know if you did something wrong. So it’s kinda like mmmm, you know…but that’s not yeah…no.

Participants noted that they found it hard to speak to friends or family who they feared wouldn’t understand the nature of their stress. Nurses were also worried about burdening others with their stress and mentioned HIPPA or privacy rules for restrictedness as it pertained to outsiders, co-workers, and subordinates. For instance, Leslie states:

*I leave work at work and home at home. Um, I don’t really know. Probably. Probably a lot has to do with HIPAA violations and I’m always very careful not to break any of the uh privacy policy for the residents. If I discuss it with another co-worker or I discuss it with my supervisor I am not breaking those HIPPA violations. Um also people at home, wouldn’t understand what I go through at work during the course of the day. Well, there’s only one person in my house. And compared to what I do, what he does seems like a whole lot worse, so…*.

Most of the nurses we spoke with reported some amount of CROS and found it to be distressing. Some expressed an additive effect of stress – keeping issues bottled up only made those issues more stressful and wanting to be able to express their feelings to others, but not being able to becomes frustrating. Others indicated that CROS made them feel as though others had a lack of empathy for them. Overall, CROS was a significant concern for the nurses in this sample. For those who were able to have someone to speak about their stress with, they reported catharsis and stress reduction because of being able to discuss their stress. For these nurses, being able to talk to others was extremely important.

#### Effort-reward imbalance and CROS

Although all the nurses in our sample reported experiencing significant stress, the majority felt that the rewards of the job either outweighed the stressors (3 participants), or that there was a balance between the amount of stress and reward (4 participants). Rewards included saving lives, the joy of seeing a patient recover or get discharged, improving patients’ quality of life, and gratitude from patients and their families. Nurses also reported a personal sense of accomplishment more generally, the value of helping someone, and being able to problem solve. Lee, a pre- and post-op nurse tells us that:

*I think sometimes the most rewarding where I’m at now is when I can help those patients that don’t have the income for like their pain medicine…. Um, to be able to help them by like finding coupons and different things and helping them make sure they have what they need to go home because we don’t always have social workers that will do that for us.*

A third of our sample reported that the rewards did not outweigh the stress, such as Orchid:

*Oh, they don’t balance out at all. Because you don’t like, you don’t have as much accomplishment, as you have kind of failures. Like you know patients they pass on, or you know, a wound that just does not heal. Or someone’s like, they may have went home, and then two weeks later they are right back for the same symptoms. So it’s not, it’s not equal, it’s really not. That’s why you kind of hold on to the few little ones, you get.*

In looking at emergent patterns, we found that the participants who expressed an imbalance were the same nurses who reported significant CROS. For instance, Willow told us that the challenges outweigh the positives in her work. This correlated with some of the more blatant examples of CROS in our sample. She says:

*I mean, because you want to be able to express how you’re feeling. Um but pretty much when you do the reaction is: “well then you’re not organizing your time appropriately.” You know, and so when somebody says that you clam up. You shut up. You know. It’s… it’s not worth going on any further, you know, because they’re not supporting you.*

Willow notes that she wants to talk about stress but management is unavailable and unapproachable. She notes that some people vent online but she is scared to do so for fear of retribution because management monitors social media use. She does feel that she can talk to family or co-workers but also feels restricted in speaking to supervisors and feels she must monitor whom she speaks with and how she communicates. This finding was consistent across participants with self-reported ERI and this is especially illuminating given the quantitative results reported earlier. Although there was variation among those who felt no ERI or a balance with respect to their experiences of CROS, all the high ERI nurses also reported significant CROS.

## Discussion

Our primary purpose was to evaluate how CROS might play a role in the experience of nursing stress, based on nurses’ reports of effort-reward-imbalance. There continues to be a need to elucidate the mechanisms by which nurses’ work environments influence outcomes such as health and productivity [40]. In addition to the high turnover rate, nurse stress also leads to medical errors, patient morbidity and mortality [2, 5], low self-esteem, and increased absenteeism [15]. The most devastating of the listed effects of nurse stress is suicide and suicidal ideation by nurses [41]. Despite abundant research on the phenomenon of nurse stress, barriers to resolving the issue of workplace stress for nurses persist. To that end, we feel that this study demonstrates that CROS can help explain how communication can function to both exacerbate and alleviate the negative postliminary effects of work stress for nurses. Our findings also allow us to make targeted recommendations for improving work conditions and ultimately for improving outcomes such as lost productivity and poor general health among nurses.

There are two key findings that are integral to this study. First, among nurses, CROS functions to serve as an effort in the effort-reward-imbalance model. This was especially true for those nurses who had low reported perceived organizational support. In this sense, nurses reported the greatest imbalance between their efforts and their rewards when they had no outlet to discuss their stressors with others and they did not feel particularly supported by their organization. The second important finding comes from our member-checking interviews, which supports and informs the quantitative findings. Nurses from our sample relied heavily on their colleagues for social support, especially when their working conditions were not good (a common experience from nurses). Those nurses who talked about the combined effects of all other stressors with limited (or no) provisions for supportive interactions (i.e., CROS) reported the greatest stress-outcomes. Taken together, our findings point to the importance of recognizing CROS as an organizational variable for nurses and underscore the importance of bolstering interpersonal communication systems in healthcare settings. Below we describe the theoretical implications of our findings, some practical implications for nurses, and some directions that we expect this research to take.

### Theoretical implications

#### CROS

It is unsurprising that the data revealed that nurses experience CROS. Organizational members may have a range of reasons for why they feel restricted in their ability to discuss their work stress. These causes of CROS include individual power dynamics, risks associated with self-disclosure, fear of conflict, fear of burdening others, and social inappropriateness. Furthermore, workers may fear partner unresponsiveness or futility of discussion, especially if the conversational partner has little familiarity with the organization or the nature of the work [26, 42, 43]. The data presented here indicate that these same factors are quite prevalent for nurses and do, as expected, contribute to CROS.

External forces may also serve to restrict communication such as organizational privacy policies (e.g., the Health Insurance Portability and Accountability Act or “HIPAA”) or other systemic prohibitions against talking to others about the workplace [24]. In some cases, workers may feel that they are comfortable speaking to members of a particular relational domain and not others (e.g., friends/family vs. co-workers, or supervisors vs. subordinates). Our data demonstrates that these dynamics, which have been explicated in the literature in a variety of professions, exist among nurses and function in expected ways for this population [24–26, 42].

Results indicated that nurses do experience CROS and that its associated distress covaries with overall nursing stress, both of which vary by nursing type. Additionally, these findings extend the previously reported explanations for how CROS functions noted above. Specifically, the subcomponents of the CROS phenomenon have differential effects. For instance, feeling restricted in one’s ability to vent frustrations may function differently to exacerbate stress than feeling as though one cannot effect change within their workplace. This proposition needs to be examined directly in future studies. However, there does appear to be preliminary evidence in our data to indicate that when CROS relates to specific workplace stressors that nurses feel stymied in their ability to change, their experiences in the workplace are worsened.

In the case of this sample, we show effects on lost productivity, insomnia, and poor general health. These findings support the contention that nurses’ outcomes are at least indirectly affected by their ability to productively discuss their stress in a way that leads to actual change. Our thematic analysis of the member-check data further explains these findings -- feeling CROS with respect to feeling empowered to make change (such as with managers and other superiors), resulted in increased distress among nurses.

We also were able to show that CROS distress is inversely related to organizational support which is consistent with prior literature suggesting better outcomes in more supportive environments [44]. These findings support existing theorizing [24], suggesting that CROS exerts its negative effects by preventing the translation of support into action and/or because one’s illusions of perceived support are shattered when they realize that they cannot enact any support they thought that they had.

We are also able to shed additional insight on research indicating that nurses in particular, struggle with work-life-integration [45]. Nurses who feel more CROS may also feel a stronger need to compartmentalize their feelings about work and therefore suffer more ill effects due to a lack of support. The interview findings highlight the anguish nurses feel when they are unable to discuss their work stress with family or friends because they worry that they are not allowed to, that they will not be understood, or because they simply do not want to burden others with their problems. This leads to the feeling that others lack empathy for them and of internalization of their problems.

We should note that these results reflect the nature of nursing work prior to the start of the COVID-19 pandemic. More recent work suggests that these issues not only have not gone away but have increased, as Craw and colleagues [19] discovered. They found that nurses reported greater social pressures at work, compounded by the complexities of managing the stress associated with emergency care during the pandemic. In fact, through their interview study with working nurses, Craw and colleagues [19] explicated a primary theme that emerged among COVID-19 nurses -- that “nobody else understands.” The nurses they interviewed reported challenges with communicating to family members and friends about their stressful experiences all the while struggling to communicate with fellow nurses, as they were all experiencing similar stressors [19]. In these cases, nurses felt a sense of CROS and are prevented from fully utilizing their social support networks. As such, we believe the findings presented herein are especially important to consider given that working conditions have become even more stressful for nurses over time.

In previous work [24, 25] the general existence and prevalence of CROS has been identified. Subsequent work has shown that CROS is found in a variety of organizational settings such as the Catholic Church [46], among university faculty [26] and graduate teaching assistants [42], and can help in the theoretical explanations for both the structure and function of workplace stress. The present study extends those findings by examining prevalence and effects of CROS in a specific occupational subcategory that we expect to be particularly affected by this phenomenon. Given the life-or-death consequences associated with stress among nurses [19, 44, 47–49], we felt that examining CROS in this population was important.

#### ERI

Effort reward imbalance is an insidious construct in organizational stress research [50], as when chronic, the feeling of imbalance becomes a toxic feature of a worker’s organizational experience [21]. Perceived imbalance is linked to specific neurological and neuroendocrine responses. “The recurrent experience of failed reciprocity is expected to afflict the health and well-being of working people by compromising their self-esteem and by eliciting negative emotions with special propensity to elicit sustained autonomic and neuroendocrine activation of the organism” [22]. For instance, researchers evaluating physiological data from the famed Whitehall II study found that ERI was associated with waking cortisol profiles [23]. Furthermore, a 52-country epidemiological study with nearly 30,000 participants found that coronary heart disease was elevated among individuals with high efforts and low rewards at work [51]. When examining nurses specifically, Bakker and colleagues [20] found that those nurses who reported a greater imbalance (high efforts to low rewards) also reported greater levels of burnout. Furthermore, their data also revealed a moderating effect of intrinsic effort (or need for control), where burnout was highest among those nurses who reported an imbalance and a need for control. This is important for the present investigation, as reciprocity in the support dynamic is an element in nurses’ reported need for control.

While conventional thinking might view explicit organizational stressors (e.g., long working hours, dangerous working conditions, etc.) as being prevalent efforts, we believe that other more implicit variables also play an important role in understanding the full scope of efforts. Our findings support the notion that CROS is likely an effort in this model. This is important, as CROS is an interactive-communicative variable – as described earlier, CROS prevents a person from potentially enacting support structures or engaging their own support schemas. While other theorists have connected a variety of psychological constructs to ERI [20, 21, 23], we are the first to demonstrate how CROS fits into this model.

When considering the deleterious effects of ERI, we found that CROS serves to amplify the effects – those nurses who reported the highest amount of CROS stress with the lowest amount of organizational support and the highest levels of ERI had the highest amounts of nursing stress. When CROS distress was low, organizational support was high, and ERI was below 1.00 (indicating more rewards compared to efforts), nursing stress was the lowest. These quantitative findings were echoed among the nurses we interviewed. Taken together, we believe that, for these nurses, CROS was a meta-stressor [24] that served to amplify the experience of nursing stress, since it functioned as an organizational effort.

Importantly, our study specifically focuses on nurses’ reports of ERI. Nurses experience a variety of stressors as a function of their work environment and occupations [17, 52] and their relationships with patients and coworkers [44]. For nurses, ERI predicts burnout [20]; therefore, it was no surprise that we found that the nurses in our sample also experienced ERI. Additionally, nurses in our sample reported negative associations between ERI and organizational support and perceived general health, and ERI was positively associated with insomnia and productivity lost. These results paint a troubling picture for nurses who report high efforts, low rewards, high levels of insomnia, lower productivity, lower organizational support, and lower perceived general health.

Ultimately these findings help extend our understanding of how ERI and CROS function in this context and provides a more robust explanation for how downstream negative effects (such as lost productivity and insomnia) are amplified in the nursing profession. Put simply, not having the ability to garner support through communicative channels in a high stress situation with low organizational support can lead to an imbalance in how rewarding work is. This is important, as the nurses we spoke with told us that their reason for getting into the nursing profession was to help people – their reward was intrinsic. An exacerbation of ERI can have a devastating effect on a nurse’s work-life experiences and their vocational identity. The results from this study indicate that for many nurses feeling communicatively restricted lessens their perceptions of rewards and/or increases the burdens or efforts associated with the work. For many nurses, the intrinsic reward associated with nursing was outweighed by the costs. That imbalance has the potential for a wide variety of negative stress-related outcomes. For these reasons, we believe that a focus on the practical implications is warranted.

### Practical implications

Taken as a whole, we can explain a mechanism by which communication plays a role in how nurse stress influences nurses’ lived outcomes. Our data allowed us to reflect the experiences of a range of individuals across demographic categories and from a representative range of nursing professionals. As such, we are confident that these findings generalize to nurses in the US, providing valuable insight into this population. Given the importance of addressing nurse stress outlined above, we believe this to be a significant contribution to the body of knowledge on nursing.

Per National Institutes of Occupational Safety and Health recommendations, alleviating work stress often requires change on the part of organizations. However, the wholesale changes and cultural shifts advocated by scholars have not been readily embraced [53]. While many organizations provide access to employee assistance programs, data suggest that they are underutilized [54] and, individuals tend to be hesitant to seek formal mental health care [55]. As a result, workers are often left to rely on informal social support to address their workplace stress [56–59]. However, sufficient social support is not always readily available.

Recognizing the systemic communication issues that nurses face in the workplace can lead to avenues for significant change. Furthermore, we suggest that interventions aimed at reducing CROS could reduce ERI. The reduction of ERI would lead to improvements in downstream health and psychological outcomes for workers [20, 50, 60, 61]. To the extent that communication is a major stressor that is moderated by CROS, targeted interventions aimed at improving communication can be cost efficient mechanisms for reducing the negative stress-related outcomes we see for nurses and in nursing organizations. For instance, organizations that support appropriate levels of open communication within the organization can (1) reduce CROS, and (2) improve nurses’ perceptions of support therein affecting two of the variables that lead to negative outcomes [48]. Furthermore, in a study of 201 hospital nurses, Apker and colleagues [62] discovered that nurses who co-create synergistic team communication systems are less likely to leave their organizations, thereby demonstrating the importance of high-quality team-based communication in reducing turnover. Thus, we encourage management to actively cultivate an environment where nurses do not feel restricted in their ability to discuss concerns. To the extent that our findings suggest that futility of communication is especially distressing, we feel that it can be particularly useful for organizations to consider open communication policies. Furthermore, managers should act on issues nurses communicate to them, thereby addressing both the underlying stressors and the exacerbating effects of restricted communication pertaining to those stressors. This two-pronged approach can lead to the most marked improvements.

Although communication that leads to change can be transformative, simply being receptive to open discussion of stressors or job efforts can also in and of itself also help alleviate some of the negative effects associated with nursing work. We are not the first to note the importance of communication in improving nurses’ workplace environments [2]. For instance, a recent white paper indicated that when it comes to ways mental health employees suggest reducing job related burnout, the two most common themes in the responses were staff camaraderie and personal connections with co-workers, and the development of a culture of openness to acknowledge and discuss burnout [63]. Other research also shows that workplace support for nurses is associated with decreased burnout and desire to quit [2]. In our own findings, we saw that those who reported less CROS, reported experiencing catharsis and stress reduction by virtue of their sense of open access to supportive communication and lack of restrictedness. In sum then, these findings are supportive of organizational changes that focus specifically on communication processes that reduce CROS and improve organizational support.

### Limitations and directions for future research

This project affords insight into the role of CROS within the ERI framework for nurses and allows us to make specific recommendations for changes that can improve outcomes of interest like nurse stress and productivity. However, some limitations must be noted. First, we acknowledge that data were collected cross-sectionally and at a particular point in time. Although the interview data provided support for the logic of our conclusions, any causal claims would need to be explored further using longitudinal methods. While our dataset did include good diversity with respect to nursing type, we did not see much ethnic or racial diversity. Therefore, replicating this work with a larger and more diverse sample might bolster our claim of generalizability and can elucidate nuances in experiences that exist within subpopulations of nurses. Furthermore, given that the global pandemic has fundamentally altered the nature of healthcare work, additional work on how CROS functions in a post-COVID world may be needed to fully understand nurses’ experiences and we believe this to be an important avenue for future research. Finally, we believe that investigating the ethical obligations of organizations in reducing ERI and CROS would be valuable. Given that ERI has been framed as a form of organizational injustice [64], it stands to reason that addressing CROS can make for a more just organizational climate. Such positioning should be investigated in future work.

## Conclusion

Communicatively Restricted Organizational Stress (CROS) is a meta-stressor that leads to a constellation of negative individual level outcomes for organizational members such as poor health or reduced work capacity. The results of the present study indicate that for working nurses, CROS functions as an effort in the Effort-Reward-Imbalance (ERI) framework and thus contributes specifically to productivity lost, insomnia, and decreased general health. Individuals with low levels of organizational support and high levels of CROS, reported the highest levels of ERI when nurse stress was high. Findings also indicate that CROS is prevalent among nurses and its associated distress can differ by nursing type with nurses working in mental health experiencing the most CROS and nursing stress, and home health nurses reporting the least. Follow-up interviews confirmed that stress is universal for nurses and is a result of workload, communication issues, bureaucracy, and patient/family interactions. CROS experiences vary substantially and can be readily identified and described by nurses. Finally, consistent with the quantitative findings, nurses’ reports of high subjective ERI co-occurred with significant experiences of CROS. Taken as a whole, this project contributes to a theoretical understanding of both CROS and ERI as organizational phenomena and allows us to make recommendations for improving organizational experiences for nurses. We especially suggest cultivating a culture of open communication, acting on nurses’ concerns, improving nurses’ perceived agency to effect change, reducing silencing, and introducing opportunities for increased camaraderie among employees.

## Data Availability

The dataset used and/or analyzed during the current study is available from the corresponding author on reasonable request.
